# Relationship of postsaccadic oscillation with the state of the pupil inside the iris and with cognitive processing

**DOI:** 10.1152/jn.00205.2019

**Published:** 2019-12-11

**Authors:** Shimpei Yamagishi, Makoto Yoneya, Shigeto Furukawa

**Affiliations:** NTT Communication Science Laboratories, Kanagawa, Japan

**Keywords:** postsaccadic oscillation, pro- and antisaccades, pupil, saccade dynamics

## Abstract

Recent studies using video-based eye tracking have presented accumulating evidence that postsaccadic oscillation defined in reference to the pupil center (PSOp) is larger than that to the iris center (PSOi). This indicates that the relative motion of the pupil reflects the viscoelasticity of the tissue of the iris. It is known that the pupil size controlled by the sphincter/dilator pupillae muscles reflects many aspects of cognition. A hypothesis derived from this fact is that cognitive tasks affect the properties of PSOp due to the change in the state of these muscles. To test this hypothesis, we conducted pro- and antisaccade tasks for human participants and adopted the recent physical model of PSO to evaluate the dynamic properties of PSOp/PSOi. The results showed the dependence of the elasticity coefficient of the PSOp on the antisaccade task, but this effect was not significant for the PSOi. This suggests that cognitive tasks such as antisaccade tasks affect elasticity of the muscle of the iris. We found that the trial-by-trial fluctuation in the presaccade absolute pupil size correlated with the elasticity coefficient of PSOp. We also found the task dependence of the viscosity coefficient and overshoot amount of PSOi, which probably reflects the dynamics of the entire eyeball movement. The difference in task dependence between PSOp and PSOi indicates that the separate measures of these two can be means to distinguish factors related to the oculomotor neural system from those related to the physiological states of the iris tissue.

**NEW & NOTEWORTHY** The state of the eyeball varies dynamically moment by moment depending on underlying neural/cognitive processing. Combining simultaneous measurements of pupil-centric and iris-centric movements and a recent physical model of postsaccadic oscillation (PSO), we show that the pupil-centric PSO is sensitive to the type of saccade task, suggesting that the physical state of the iris muscles reflects the underlying cognitive processes.

## INTRODUCTION

The state of an eyeball varies dynamically moment by moment, reflecting the activity of various muscles, namely, extraocular muscles (controlling gaze direction), sphincter/dilator pupillae muscles (controlling pupil diameter), and ciliary muscles (controlling lens thickness). The primary functions of these muscles are to regulate the visual information coming into our eyes (e.g., to adjust the light intensity or maintain a clear image). The state of an eyeball is also known to reflect various aspects of perception, attention, and cognition, which are not directly related to visual signal processing (Hynes et al. 2018; Mathôt 2018; Rolfs et al. 2005).

Previous studies found that the postsaccadic oscillation (PSO), or dynamic overshoot (Bahill et al. 1975), of the pupil-centric trajectory was larger than that of the iris-centric trajectory or a search coil signal (Kimmel et al. 2012; Nyström et al. 2013). It is possible that iris-centric or search coil signals reflect predominantly the rigid movements of the entire eyeball, whereas the pupil-centric signals reflect the viscoelastic movements of the tissue inside the iris (if the oscillation becomes overt, it is called “iridodonesis” in the clinical field; Desai and Tajik 2017). This possibility is supported by a recent study, which demonstrated that a physical model incorporating the dynamic movement of the viscoelastic iris can account for the experimental observation (Bouzat et al. 2018).

The states of the sphincter/dilator pupillae muscles, which control pupil size, may affect the viscoelastic states of the iris. It is fair to assume that the state of pupillae muscles covaries with the physical properties of the iris because the iris tissue embeds these muscles. Thus the state of pupillae muscles may determine the dynamic properties of PSO (cf. Nyström et al. 2016). This hypothetical relationship leads us to expect that the cognitive states of individuals are reflected in the PSO properties, because pupil size reflects many aspects of cognition such as processing load, attention, and preparation of eye movements (Mathôt 2018).

This study tested this expectation by examining whether/how the cognitive task affects the properties of PSO, measured as pupil-centric and iris-centric trajectories captured by a high-speed camera. We focused on iris movements defined by the pupil center derived from the pupil-iris border (denoted as “pupil centric”) and the iris center derived from the scleral-iris border (denoted as “iris centric”). The PSOs of the pupil-centric and the iris-centric movements are denoted as “PSOp” and “PSOi,” respectively. The viscoelastic movement of the iris tissue is called “iris wobble.” We adopted the physical model of Bouzat et al. (2018) to treat the PSOs parametrically.

To evaluate the contributions of cognitive states, we examined the properties of PSOp/PSOi during pro- and antisaccade tasks (Munoz and Everling 2004). The participant is instructed to make a saccade toward (prosaccade) and away (antisaccade) from a cued location as fast as possible. We considered these tasks to be in line with the aim of this study, because they involve saccades and the cognitive effect simultaneously. The antisaccade task has been widely used to investigate the nature of voluntary behavior. The saccadic reaction time is longer for an antisaccade compared with a prosaccade, because the execution of the antisaccade requires the top-down inhibition of an prosaccade (Munoz and Everling 2004). This inhibition is considered to be mediated by cortical projections to the intermediate layer of the superior colliculus (SCi): patients with neurological diseases involving the frontal cortex or the basal ganglia have difficulties suppressing prosaccade toward stimulus during an antisaccade task (Guitton et al. 1985; Peltsch et al. 2008). A recent study found that the change in presaccade pupil size was larger during antisaccade preparation than during prosaccade preparation (Wang et al. 2015). The study suggested that the preparatory activity of SCi is reflected in pupil size through the link between SCi and the pupil control circuit (Wang et al. 2015; Wang and Munoz 2015). Given this fact, we can expect a link between PSOp and the presaccade pupil size during an antisaccade task.

## MATERIALS AND METHODS

### 

#### Participants.

Eighteen adults (2 men and 16 women) participated in the experiments. Their ages ranged from 22 to 52 yr (mean = 38.2 yr). The experimental protocols were approved by the Research Ethics Committee of Nippon Telegraph and Telephone (NTT) Communication Science Laboratories. All participants gave written informed consent before the experiment.

#### Apparatus.

The data were recorded with a high-speed camera (IDP-Express R2000; Photoron, Tokyo, Japan) at a sampling rate of 500 Hz. The recorded images were eight-bit grayscale images with a resolution of 512 × 256 pixels. Visual stimuli were generated with MATLAB (R2016b) and were presented on a monitor with a resolution of 1,920×1,080 pixels and a refresh rate of 60 Hz. The participants sat on a chair, and their heads were stabilized with a chin rest. The distance between the chin rest and the center of the display was 70 cm. Auditory stimuli were synthesized with MATLAB at a sampling frequency of 44.1 kHz and were presented through a digital-to-analog converter and headphones (MDR-7506; SONY, Japan).

#### Stimuli and procedure.

**Fig. 1.**
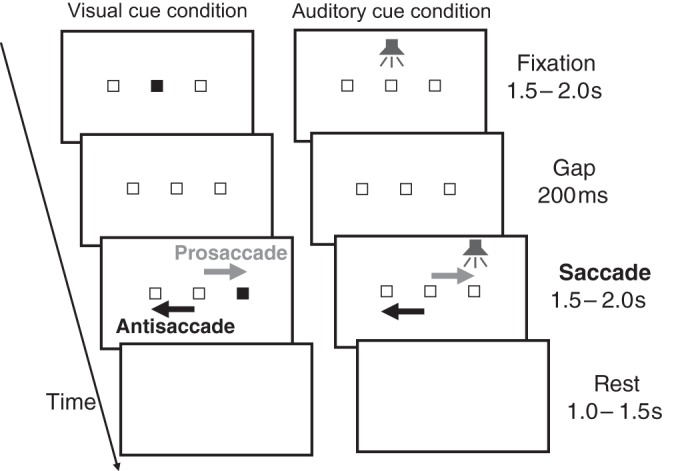
Schematic representation of pro- and antisaccade tasks. In the prosaccade task, participants were asked to make a saccade in the direction toward a peripheral visual cue or auditory cue. In the antisaccade task, they were asked to make a saccade in the direction away from the visual cue or auditory cue.

In *experiment 1*, participants were engaged in four types of tasks that differed in the cue modality that guided the saccade (visual vs. auditory cues) and in the direction of the saccade relative to the target (pro- vs. antisaccades), namely, visually guided prosaccade (V_pro), visually guided antisaccade (V_anti), auditorily guided prosaccade (A_pro), and auditorily guided antisaccade (A_anti). The four tasks were conducted in separate blocks of trials. The task order was V_pro, V_anti, A_pro, and A_anti for all participants. Figure 1, *left*, shows the stimulus presentation sequence in one trial of the visual cue condition (i.e., V_pro or V_anti tasks) in *experiment 1*. The luminance of the background of the display was 149 cd/m^2^ throughout the experiment. This experimental paradigm was designed with reference to Walker et al. (2000); i.e., visual landmarks (empty squares) for peripheral positions were displayed to make the amplitudes of the pro- and antisaccades nearly equal. It should be noted that this method of stimulus display is somewhat different from that in typical antisaccade experiments, in which the display has a totally dark background and no landmark (Munoz and Everling 2004); therefore, caution is needed when the current results are compared with those of earlier studies. The existence of a landmark, for instance, could affect prosaccade performance to some extent. Nevertheless, we believe that this difference had little impact on the interpretation of the present study, at least on the cognitive processes underlying saccade behavior: as described in results, the difference between pro- and antisaccades was still significant (Briand et al. 1999). Regarding the background luminance, Pratt and Trottier (2005) reported a similar pattern of saccadic reaction time for both white (32 cd/m^2^) and black background (~0 cd/m^2^).

At the beginning of a trial, the participant was asked to fixate on a filled square (1° each side) at the center of the display, ignoring the two empty peripheral boxes on the left and right of the filled square, for 1.5–2 s (fixation period). The filled square then changed to an empty square for a period of 200 ms (gap period), followed by one of the peripheral squares changing to a filled square. The luminance of the filled square was 2.49 cd/m^2^. The peripheral square remained filled for 1.5–2 s (saccade period). In the prosaccade task, the participants were asked to make a saccade in the direction of the filled square. In the antisaccade task, the saccade direction was opposite. After the saccade period, all the visual stimuli disappeared (rest period: 1–1.5 s). The participants were asked to try not to blink during the fixation period. All the participants took part in 60 trials for both the pro- and antisaccade tasks. Data were recorded only for the first 1.5 s of the fixation and saccade periods (3 s for 1 trial) due to the limit of recording buffer of the high-speed camera.

In the auditory cue condition (Fig. 1, *right*), an auditory cue was used instead of a visual cue for the saccade initiation. The fixation and saccade directions were indicated by the white noise (A-weighted sound pressure level: 70 dB) presented from the center of the head or left/right ear, respectively. This condition was examined to evaluate the effects of the difference in the retinal inputs between the V_pro and V_anti conditions. The procedure was the same as the visual cue condition, but all the visual stimuli were empty squares and were constant throughout the fixation and saccade periods.

We conducted a control experiment (*experiment 2*) to confirm whether the gaze-dependent distortion in a camera image can affect the estimation of the PSO. This concern arises from the fact that the iris size is always larger than the pupil size, suggesting that the iris center position may be underestimated compared with the pupil center position on the camera image axis. We expected this distortion to increase with increasing saccadic amplitude. Thus we tested prosaccades toward a target with various saccade amplitudes (−13, −9, −5, −1, 1, 5, 9, 13°). The procedure of the task was similar to the main experiment (Fig. 1). All the participants took part in 12 trials for each target position, and the order of target position was randomly chosen. The participants were the same as in *experiment 1* except that one participant from *experiment 1* did not take part in *experiment 2*. In this experiment, we used only a visual cue to guide the saccade. We excluded all the trials in the condition of the absolute saccade amplitude of 1° because there were few valid trials after data screening.

#### Detection of pupil and iris centers.

**Fig. 2.**
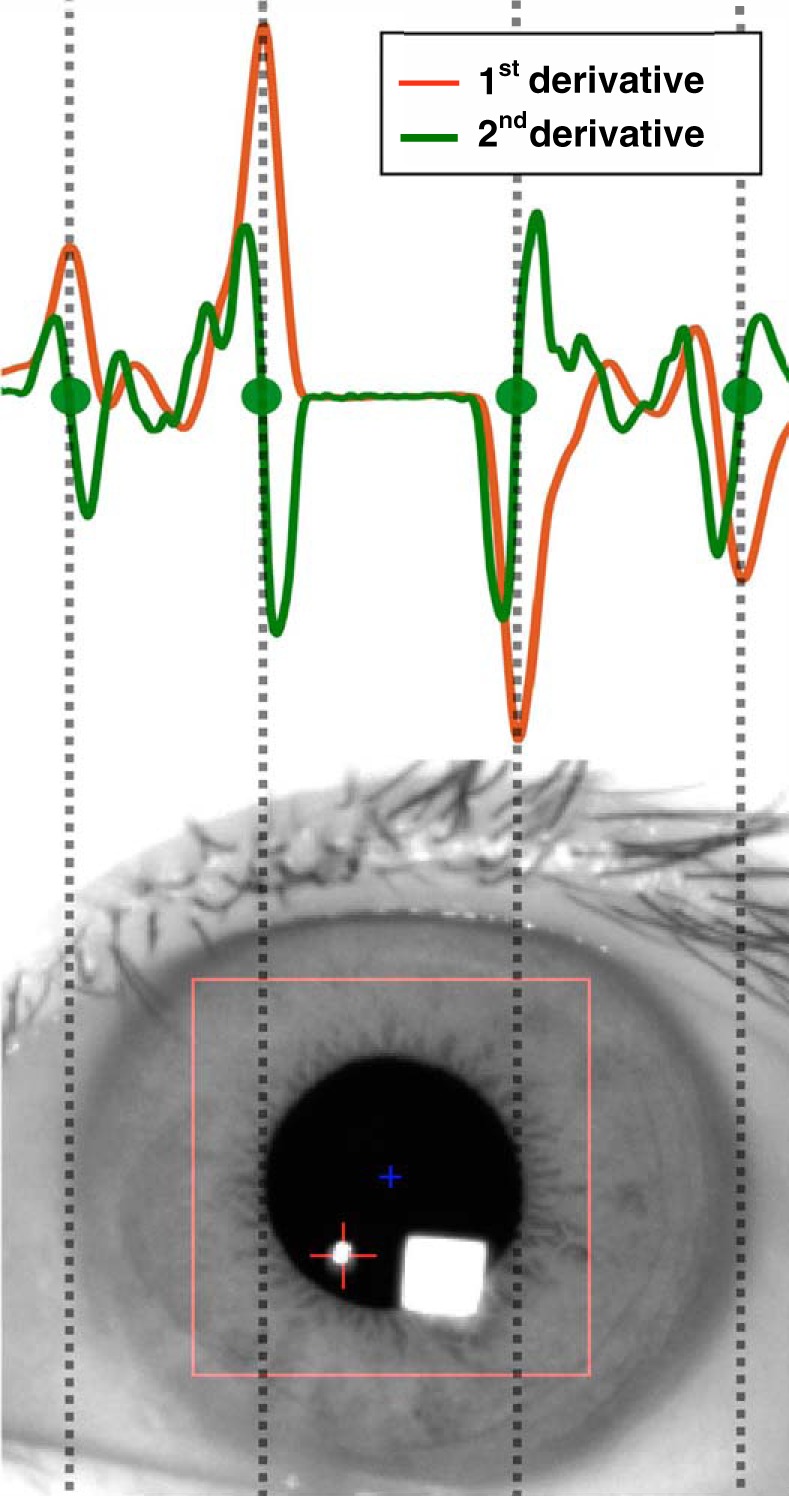
Example of edge detection of pupil and iris. First, the provisional position of the pupil center was derived as the center of gravity of the pupil area (blue cross in image). Second, edges were roughly estimated by finding the positive and negative peaks of the 1st derivative of the gray value (orange line). Next, the 2nd derivative of the gray value (green line) was calculated. The subpixelized edges of the pupil and iris were then determined as the zero-cross point of the 2nd derivative of the gray value around the peaks of the 1st derivative (green circles).

First, we extracted a pupil area whose intensity was less than a certain threshold value (15–40). The threshold was determined for each subject because the light condition and gray value of the pupil were slightly different among subjects. The provisional position of the pupil center was derived as the center of gravity of the pupil area. Second, the gray value along a horizontal line was calculated by averaging two rows of pixels above and below the provisional pupil center. Third, the peaks of the first derivative of the horizontal gray value were extracted. The first derivative of the gray value was calculated simply by subtracting neighboring values with an averaging window of 10 pixels. Fourth, we defined the subpixelized edges of the pupil and iris as the zero-cross points of the second derivative of the gray value. The zero-cross points were determined by searching around the peaks detected in the third step (Fig. 2). Finally, the pupil and iris centers were defined as the midpoints of the pupil and iris edges, respectively. We then obtained a time series of pupil and iris center trajectories. We treated data with no pupil or iris edges as missing data (caused by blinking or unsuccessful edge detection).

#### Detection of saccades.

For each participant and condition, the velocity of the displacement of the pupil/iris centers was calculated by using an averaging time window of two frames. The onset of a saccade was defined as the time point at which the velocity exceeded a certain threshold. The velocity detection threshold was 8σ, where σ represents the standard deviation of velocity in one condition. For each trial, we analyzed only the first saccade during the saccade part. If we detected saccades with amplitudes that were obviously smaller than the expected value (9° in viewing angle, 20–25 pixels in camera axis), we discarded these from our analysis as microsaccades, and we adopted the next saccade for analysis.

#### Model of saccadic eye movement.

To assess the overshoot property of saccadic eye movement, we used the differential equation of forced oscillation proposed by Bouzat et al. (2018):

(1) $$\ddot{y}+\gamma \dot{y}+ky=-\ddot{x}$$

where *x* is the position of the cornea (eyeball), *y* is the relative position of the pupil center measured from *x*, γ is the viscosity coefficient, and *k* is the elasticity coefficient. Viscosity is the resistance of liquid-like objects to flowing. For example, water flows easily and has a small viscosity coefficient, whereas honey has a large viscosity coefficient and spends more time flowing compared with water. The viscosity coefficient may reflect how large the peak dynamic overshoot (overshoot amount) is. Elasticity is the resistance of the solid-like objects to an applied force. It represents the ability of the body to return to its original size and shape when an applied force is removed. For example, a rubber ball is easy to deform and has a small elasticity coefficient, whereas a metal ball is hard to deform and has a large elasticity coefficient. The elasticity coefficient of PSO may reflect how large the oscillation or deformation of iris tissue is. Overall, a larger viscosity coefficient or elasticity coefficient indicates a larger viscous resistance or elastic resistance, respectively, which leads us to expect a smaller PSO. At present, however, it is difficult to attribute a derived viscosity coefficient or elasticity coefficient to the material properties of particular objects (for instance, the pupil is a “hole” and actually has no mass), although they provide useful insights into the mechanisms underlying PSO.

The right side of *Eq. 1* represents the inertial force felt by the iris (or pupil-iris border) during eyeball rotation. Bouzat et al. (2018) considered the external force that represents the action of the muscles for eyeball rotation as

(2) $$\nu \dot{x}=F\left(t\right)$$

where ν is the viscosity coefficient acting on the eyeball. As in the Bouzat et al. study, we fixed ν = 1 for simplicity. The forcing function *F*(*t*) is the same as in their study and is given by

(3) $$F\left(t\right)=At\;\text{exp}\left(-\frac{t^{2}}{\tau^{2}}\right)$$

where *A* represents the global strength of the force, *t* represents a time, and τ is a time constant. From *Eq. 2* and *Eq. 3*, we can describe the eye position as follows:

(4) $$x\left(t\right)=\frac{1}{2}A\tau^{2}\left[1-\text{exp}\left(-\frac{t^{2}}{\tau^{2}}\right)\right]$$

With *Eq. 4*, we numerically solved the differential equation (*Eq. 1*). The saccade amplitude can be described by τ and *A* [$\underset{t\to \infty }{\lim\;}x\left(t\right)=A\tau^{2}/2$].

Bouzat et al. also introduced the factors (*c*,*d*), which can control the eyeball-iris link depending on the force

(5) $$\gamma =\gamma_{0}\;exp\left[-cF\left(t\right)\right],\;c>0$$

(6) $$k=k_{0}\;exp\left[-dF\left(t\right)\right],\;d>0$$

The model is described in detail in Bouzat et al. (2018).

In this study, a saccade with viscoelastic oscillation, i.e., *x*(*t*) + *y*(*t*), was fitted to the observed data of both pupil-centric and iris-centric trajectories. Although our expectation was that the PSOp would exhibit larger coefficients of viscoelasticity than the PSOi, the observed data showed that the iris-centric trajectory also exhibited PSO as well as pupil-centric trajectory. The PSOi may reflect the oscillation of the whole eyeball (Bahill et al. 1975). More specifically, the assumption by Bouzat et al. that the eyeball position *x*(*t*) in *Eq. 2* behaves in an overdamped way might be incorrect. We consider *x*(*t*) to be an ideal eye position, and the iris center is also placed on a non-inertial frame. Thus we decided to apply the above model to both pupil- and iris-centric trajectories independently and to compare the optimized parameters that represent viscosity or elasticity (γ_0_,*k*_0_).

We also evaluated the PSO more straightforwardly by calculating the amount of the overshoot in the fitted curve for each trial as follows: *1*) we calculated the peak value (*p*_1_), the local minimum next to it (*p*_2_), and the local maximum next to it (*p*_3_). *2*) We fitted the line through *p*_1_ and *p*_3_. *3*) We calculated the value on this line at the time of *p*_2_. The difference between *p*_2_ and this value was defined as the overshoot amount (*inset* of Fig. 4*B*). This procedure was the same as that employed by Hooge et al. (2015).

We also used the curve fitting method to screen out candidate saccades with irregular trajectories on the basis of a quantitative criterion. The coefficient of determinant (COD) was adopted as the criterion, and trajectories with a COD < 0.98 were excluded from the analyses. We chose this criterion value arbitrarily based on examinations of the distribution of the CODs for all saccades. Only 92 trials were excluded even with this severe criterion (2.5% of the tested data), indicating that the curve fitting accuracy was very high. After this screening, four participants with a small number of trials (fewer than 10) were excluded from the analysis.

We determined the time range for curve fitting as follows: The curve fitting end point was 40 ms after the time at which the position reached the maximum value. This duration was chosen because a typical PSO lasts 20–30 ms after the peak position (Nyström et al. 2013; Van Gisbergen et al. 1981) and a corrective secondary saccade often occurs with a very short latency after the oscillation period (Ohl et al. 2011). For each trial, the start point of the model fitting was shifted from the saccade onset in the sample range from −3 to 1 (−6 to 2 ms), and, of the five start points, we used the one that provided the highest COD in the curve fitting for each trial.

#### Evaluation of pupil size.

In the present study, we were interested in the pupil size before or during a saccade and its effects on the physical properties of the eye, which in turn might affect or interact with PSOp. We should be cautious about the gaze direction-dependent distortion of the pupil size in the video image (Choe et al. 2016; Hayes and Petrov 2016; Wyatt 2010). To factor out the distortion and evaluate the “true” variation in the pupil size, we represented pupil diameter as the ratio relative to the iris diameter. This is because we expected the apparent size changes of the pupil and iris to covary with changing eye-gaze angles, and thus the ratio should be relatively independent of eye-gaze angle, when the iris size is constant over time. Hereafter, the pupil size relative to the iris size is referred to as “normalized pupil size.”

## RESULTS

### 

#### Saccadic reaction time and error rate.

**Fig. 3.**
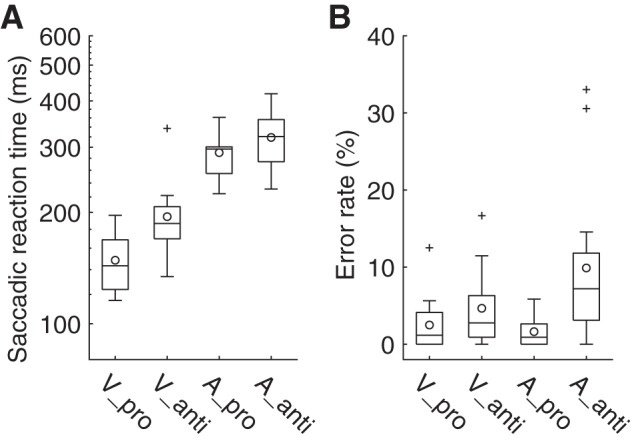
Comparison of the saccade reaction time (SRT) and error rate. Distribution of the results is represented by boxplots with the following conventions: upper and lower ends of a box and the horizontal bar in a box are the 75th and 25th percentiles and the median, respectively. Upper and lower ends of a whisker are maximum or minimum values excluding the outliers, respectively. Circles in boxplots represent mean values, including the outliers. Outliers (crosses) were defined as values larger or smaller than the 75th percentile + 1.5 × interquartile range (IQR; difference between 75th percentile and 25th percentile) or the 25th percentile − 1.5 × IQR, respectively. *A*: SRTs for pro- and antisaccades. Generally, the SRT was longer for the antisaccade than for the prosaccade, and longer for an auditorily guided saccade than a visually guided saccade. *B*: error rates for pro- and antisaccades. Error rate was higher for the antisaccade task than for the prosaccade task. Erroneous saccades more frequently occurred for the auditorily guided antisaccade (A_anti) than for the visually guided antisaccade (V_anti). A_pro, auditorily guided prosaccade; V_pro, visually guided prosaccade.

Generally, the saccadic reaction time (SRT) was longer for the antisaccade task than for the prosaccade task and was longer for the auditory cue condition than for the visual cue condition (Fig. 3*A*). A two-way repeated-measures ANOVA (task: pro vs. anti, cue modality: vision vs. audition) showed a significant main effect of task [*F*(1,13) = 19.86, *P* = 0.0006, partial η^2^ = 0.60] and cue modality [*F*(1,13) = 136.03, *P* = 0.0001, partial η^2^ = 0.91]. The effect of task on visual cue condition was as expected given the previous studies indicating that the neural circuit for antisaccade involves higher cognitive areas, such as the frontal eye field and the basal ganglia, than prosaccade (Guitton et al. 1985; Olk and Kingstone 2003; Peltsch et al. 2008). It should be pointed out that the SRT difference between the pro- and antisaccade tasks was also observed in the auditory cue condition, which is consistent with an earlier report (Heath et al. 2016). This implies that higher cognitive processes are involved in the antisaccade task than in the prosaccade task, even when the saccade is guided by auditory cues. It should be noted that the SRT difference between visual and auditory cue conditions can be accounted for by the costs associated with vector transformation from auditory space information to visual space information (i.e., head-to-retinocentric coordinate transformation) (Heath et al. 2016). Therefore, the task difficulty with the visual and auditory cue conditions may differ. This was supported by the error rate result in the antisaccade task (Fig. 3*B*). A two-way repeated ANOVA for the error rate revealed not only significant main effects of task [*F*(1,13) = 13.17, *P* = 0.0031, partial η^2^ = 0.50] and cue modality [*F*(1,13) = 4.95, *P* = 0.044, partial η^2^ = 0.28] but also a significant interaction between task and cue modality [*F*(1,13) = 6.87, *P* = 0.021, partial η^2^ = 0.35]. A postanalysis of the interaction showed that the error rate in the antisaccade task was larger for the auditory cue condition than for the visual cue condition [*F*(1,13) = 12.32, *P* = 0.0038, partial η^2^ = 0.49].

For further analysis of the properties of eye movements, we excluded trials with erroneous saccades (i.e., saccades toward the stimulus in an antisaccade condition and saccades in the opposite direction to the stimulus in a prosaccade condition).

#### Task dependence of overshoot amount.

**Fig. 4.**
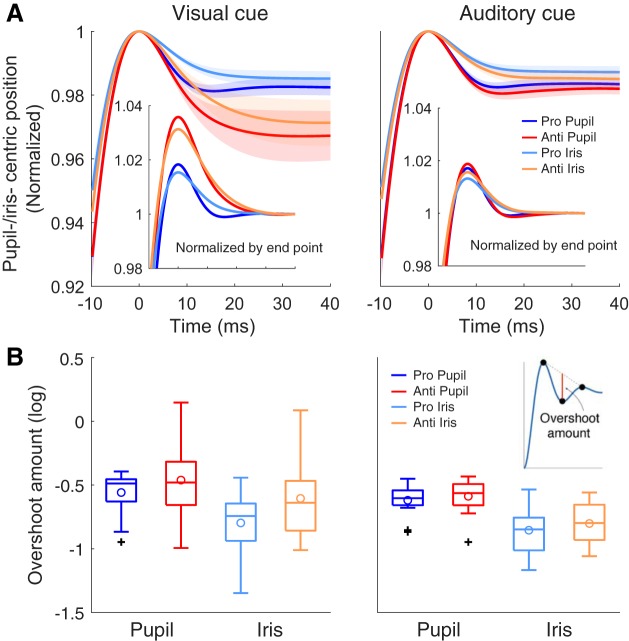
Evaluation of the postsaccadic oscillation (PSO). *A*: grand average of fitted curves. Before averaging, data were normalized by the peak of positions of the pupil-centric or iris-centric curves in the analysis range (−10 to 40 ms from saccade onset) or the saccade amplitude at the end point (40 ms from the saccade onset; *insets*). Blue and red lines show the pupil-centric curves for prosaccade (Pro Pupil) and antisaccade (Anti Pupil), respectively. Light blue and orange lines show the iris-centric curves for prosaccade (Pro Iris) and antisaccade (Anti Iris), respectively. Shaded regions indicate the range of the SE. *B*: comparison of the log-transformed overshoot amount. *Inset* at *right* represents the schematic method for calculation of the overshoot amount (for detail, see materials and methods). Generally, PSO is larger for the pupil-centric trajectory than for the iris-centric trajectory, and is larger for an antisaccade than for a prosaccade. See Fig. 3 for detailed conventions of boxplots.

First, we examined whether the dependence of task was observed for the amount of dynamic overshoot, which was calculated straightforwardly by the pupil-centric and iris-centric trajectories (see materials and methods). For each saccade, we fitted the curve described by *Eq. 1* to the pupil-centric and iris-centric trajectories (Fig. 4*A*). The trajectories shown in the Fig. 4*A* were normalized relative to the peak position or the end-point amplitude (*insets*). We performed three-way repeated-measures ANOVA (task: pro vs. anti, eye parts: pupil centric vs. iris centric, cue modality: vison vs. audition) for the overshoot amount (Fig. 4*B*). We found that eye parts had a significant main effect [*F*(1,13) = 56.39, *P* = 0.0001, partial η^2^ = 0.81, pupil centric > iris centric]. This observation that pupil-centric movement is more unstable than iris-centric movement is consistent with previous studies (Kimmel et al. 2012; Nyström et al. 2013). The fitted curve showed that the PSOi was not totally rigid (see Fig. 4*A*). This probably reflects the PSO of the entire eyeball caused by neural commands for saccade initiation (Bahill et al. 1975; Van Gisbergen et al. 1981). We also found that task had a significant main effect [*F*(1,13) = 7.33, *P* = 0.018, partial η^2^ = 0.36, pro < anti], and cue modality had a significant main effect [*F*(1,13) = 6.35, *P* = 0.026, partial η^2^ = 0.33]. The task effect implies that the overshoot amount reflects cognitive states of an individual during pro- and antisaccade tasks, and might be another way to assess the saccade behavior, as Wang et al. (2015) found in regard to pupil size.

#### Task dependence of viscoelastic parameters of postsaccadic oscillation.

**Fig. 5.**
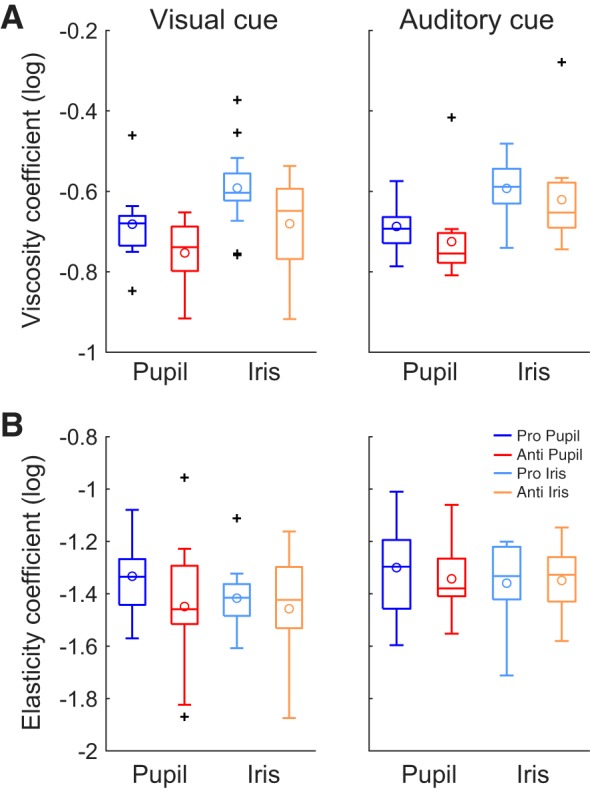
Evaluation of coefficients of viscosity and elasticity obtained by curve-fitting method. Blue and red boxplots show the coefficients of pupil-centric postsaccadic oscillation for prosaccade (Pro Pupil) and antisaccade (Anti Pupil), respectively. Light blue and orange boxplots show the coefficients of iris-centric postsaccadic oscillation for prosaccade (Pro Iris) and antisaccade (Anti Iris), respectively. See Fig. 3 for detailed conventions of boxplots. *A*: comparison of the log-transformed viscosity coefficient (γ_0_). Generally, the results for viscosity coefficient are similar to the results of overshoot amount (Fig. 4). *B*: comparison of the log-transformed elasticity coefficient (*k*_0_). Anti, antisaccade; Pro, prosaccade.

With a curve fitting method, we derived parameters (viscosity coefficient: γ_0_, elasticity coefficient: *k*_0_) that may represent the properties of iris wobble (see materials and methods). The obtained parameters are summarized in Fig. 5. To evaluate the sensitivities of those parameters to the eye parts on which movements were evaluated (pupil vs. iris), the tasks (pro- vs. antisaccades), and cue modalities (visual vs. auditory), we performed three-way repeated-measures ANOVAs independently for these parameters (Table 1). Eye parts had a significant main effect on viscosity coefficient [*F*(1,13) = 86.88, *P* = 0.0001, partial η^2^ = 0.87, pupil < iris]. Eye parts had no significant effect on elasticity coefficient (*P* > 0.1). We found that task had a significant main effect on viscosity coefficient [*F*(1,13) = 32.32, *P* = 0.0001, partial η^2^ = 0.71, pro > anti]. Generally, the viscosity coefficient exhibited similar results to those of the overshoot amount. In fact, there was a significant trial-by-trial correlation between the *z* scores of the overshoot amount and the viscosity coefficient revealed in the later analysis (Fig. 10). This means that the viscosity coefficient is associated with how large the dynamic overshoot is.

**Table 1.** Statistics of ANOVA results for PSO properties

Effect	*F* Statistic	*P* Value	Partial η2
Task			
Viscosity	**32.32**	**0.0001**	**0.71**
Elasticity	2.48	0.14	0.16
Overshoot amount	**7.33**	**0.018**	**0.36**
Eye parts			
Viscosity	**86.88**	**<0.0001**	**0.87**
Elasticity	2.87	0.11	0.18
Overshoot amount	**56.39**	**<0.0001**	**0.81**
Cue modality			
Viscosity	1.56	0.23	0.11
Elasticity	**5.81**	**0.032**	**0.31**
Overshoot amount	**6.35**	**0.026**	**0.33**
Task × eye parts			
Viscosity	0.12	0.74	0.0091
Elasticity	**23.18**	**0.0003**	**0.64**
Overshoot amount	2.02	0.18	0.13
Task × cue modality			
Viscosity	1.14	0.31	0.08
Elasticity	2.11	0.17	0.14
Overshoot amount	1.88	0.19	0.13
Eye parts × cue modality			
Viscosity	4.03	0.066	0.24
Elasticity	0.63	0.44	0.046
Overshoot amount	1.36	0.27	0.095

Data are the results of 2 (task) × 2 (eye parts) × 2 (cue modality) repeated-measures ANOVAs for 3 properties of postsaccadic oscillation (PSO): viscosity coefficient (γ_0_), elasticity coefficient (*k*_0_), and overshoot amount. Bold type indicates statistically significant properties. Degrees of freedom are omitted because all of them are the same (1,13). Three-way interactions are also omitted because they were not significant.

**Fig. 6.**
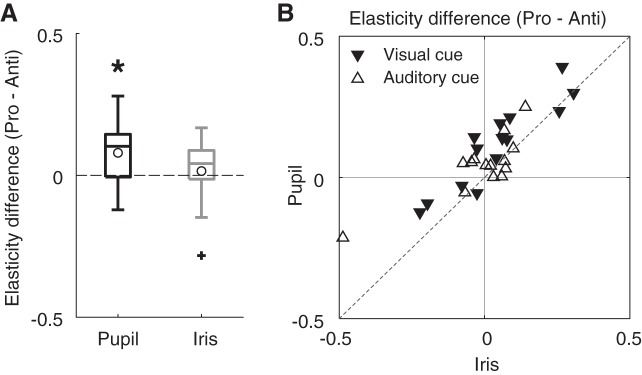
Difference in elasticity coefficient between pro- and antisaccades. *A*: black and gray boxplots represent the task differences in elasticity coefficient for pupil-centric postsaccadic oscillation (PSOp) and iris-centric postsaccadic oscillation (PSOi), respectively, which were averaged across visual and auditory cue conditions. The elasticity coefficient of PSOp is sensitive to the task (*P = 0.016), whereas that of PSOi is not (*P* = 0.65). See Fig. 3 for detailed conventions of boxplots. *B*: comparison of the task effect on elasticity (i.e., elasticity coefficient for the prosaccade condition minus that for the antisaccade condition, Pro − Anti) between PSOp and PSOi. Inverted triangles and open triangles represent individual data for visual and auditory cue conditions, respectively. Dashed line indicates unity (i.e., PSOp = PSOi).

Interestingly, there was a significant interaction between eye parts and task as regards elasticity coefficient [*F*(1,13) = 23.18, *P* = 0.0003, partial η^2^ = 0.64]. A postanalysis of this interaction revealed that the effect of the saccade task was significant for the PSOp [*F*(1,13) = 7.58, *P* = 0.016, partial η^2^ = 0.37] but not for the PSOi [*F*(1,13) = 0.21, *P* = 0.65, partial η^2^ = 0.016; Fig. 6*A*]. Figure 6*B* shows individual participant data, comparing the task effects on elasticity coefficients between PSOp (vertical axis) and PSOi (horizontal axis). The results for both visual and auditory cue conditions are represented in the same figure. The data points generally distribute above the diagonal line, indicating that the task effect tended to be larger for PSOp than for PSOi. This result might reflect the change in the state of the iris tissue (degree of iris wobble) depending on the saccade task. In addition, cue modality had a significant main effect on elasticity coefficient [*F*(1,13) = 5.81, *P* = 0.032, partial η^2^ = 0.31, vision < audition].

#### Saccade amplitude cannot account for the task dependence of viscoelastic parameters of postsaccadic oscillation.

**Fig. 7.**
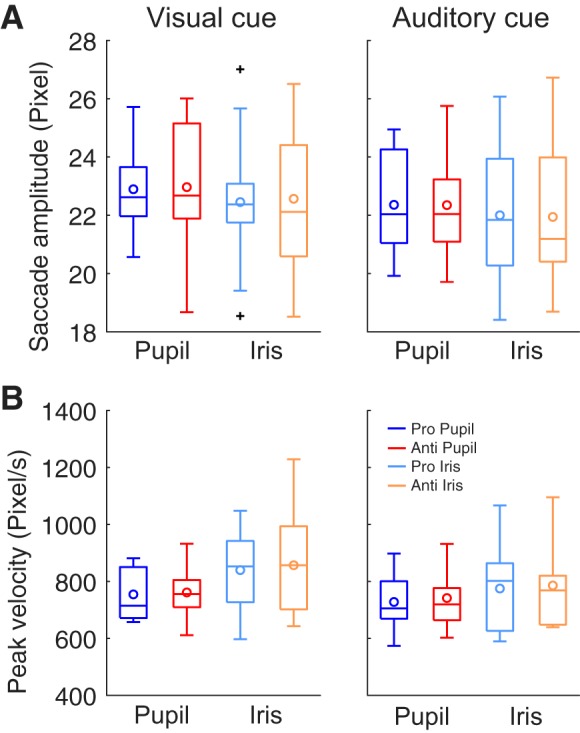
Comparisons of saccade amplitude (*A*) and peak velocity (*B*). There was no task dependence on saccade amplitude or peak velocity. Blue and red boxplots show the coefficients of pupil-centric postsaccadic oscillation for prosaccade and antisaccade, respectively. Light blue and orange boxplots show the coefficients of iris-centric postsaccadic oscillation for prosaccade and antisaccade, respectively. See Fig. 3 for detailed conventions of boxplots.

One may suspect that the above observations regarding viscoelastic parameters of PSO could be accounted for simply by the saccade amplitude at the end point of the saccades, which might depend on the task. However, we found no significant effect of task on saccade amplitude [*F*(1,13) = 0.012, *P* > 0.9; Fig. 7]. Similarly, the task had no significant effect on the peak velocity during a saccade [*F*(1,13) = 1.14, *P* > 0.3; Fig. 7).

Nevertheless, we found that cue modality had significant main effects on both saccade amplitude [*F*(1,13) = 16.07, *P* = 0.0015, partial η^2^ = 0.55, vision > audition] and peak velocity [*F*(1,13) = 9.32, *P* = 0.0093, partial η^2^ = 0.42, vision > audition]. This can be accounted for by the fact that the auditory cue provided information about the target location less explicitly than the visual cue.

Moreover, eye parts also had a significant main effect on peak velocity [*F*(1,13) = 6.34, *P* = 0.026, partial η^2^ = 0.33, pupil centric < iris centric]. This velocity difference may be due to the difference in viscosity coefficient.

#### Relationship of pupil size with task difference and postsaccadic oscillation.

**Fig. 8.**
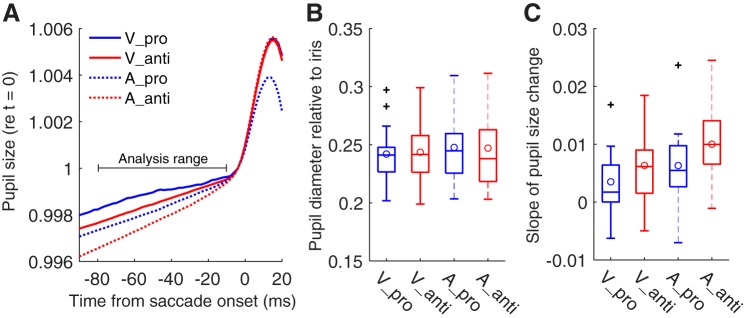
Presaccade pupil size change. *A*: time course of pupil size around saccade onset. Pupil size data were normalized by the value at the time of saccade onset (*t* = 0). Blue and red lines represent prosaccade (Pro) and antisaccade (Anti) tasks, respectively. Solid and dotted lines represent visual (V) and auditory (A) cue conditions, respectively. *B*: comparison of the presaccade absolute pupil size (average in the range from −80 to −10 ms in *A*). There were no significant differences between the 4 conditions. To factor out the distortion in pupil size, we represented pupil diameter as the ratio relative to the iris diameter (normalized pupil size). *C*: comparison of the slopes for presaccade pupil size changes (−80 to −10 ms in *A*). The pupil size change was larger for antisaccades than for prosaccades for both visual and auditory cue conditions. See Fig. 3 for detailed conventions of boxplots.

**Fig. 9.**
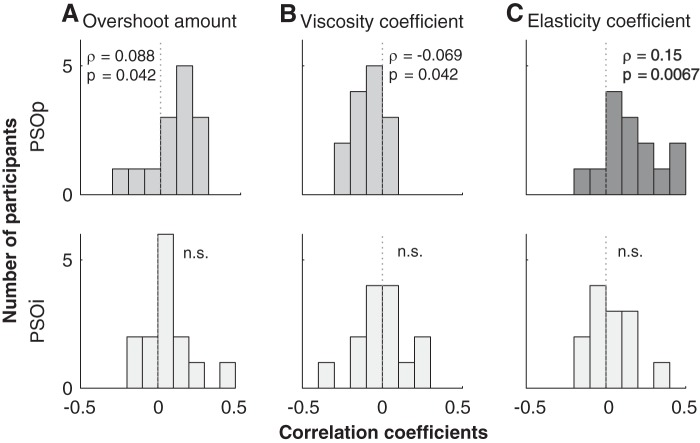
Distribution of partial correlation coefficients for the relationship between pupil size and the postsaccadic oscillation (PSO) parameters for all participants (*n* = 14). *A*: overshoot amount. *B*: viscosity coefficient. *C*: elasticity coefficient. There were significant correlations between the 3 parameters of pupil-centric POS (PSOp) and pupil size (*A–C*, *top*). The averages of correlation coefficients were ρ = 0.88 (*P* = 0.42), ρ = 0.15 (*P* = 0.42), and ρ = 0.15 (*P* = 0.0067) for overshoot amount, viscosity coefficient, and elasticity coefficient, respectively. In contrast to PSOp, there were no significant correlations (n.s.) between the 3 parameters of iris-centric PSO (PSOi) and pupil size (*A–C*, *bottom*).

The factors behind the task dependence of the elasticity coefficient of PSOp may include the physical properties of the iris muscles. It is fair to assume that pupil size is linked to the state of the iris muscles. Pupil size is known to reflect the arousal level related to the activity of the locus coeruleus-norepinephrine system (LC-NE system, Aston-Jones and Cohen 2005; Gilzenrat et al. 2010; Rajkowski et al. 1993) and also to reflect the activity of the intermediate SC (SCi) (Wang et al. 2015; Wang and Munoz 2015). Here, we shall focus on the presaccade pupil-to-iris diameter ratio, referred to as the “normalized” pupil size (see materials and methods for the reason why we used this ratio). We averaged the normalized pupil size in the −80- to −10-ms range in the time course shown in Fig. 8*A* in a trial-by-trial manner. When calculating the time course of the normalized pupil size (Fig. 8*A*), we interpolated the missing data (due to blink or unsuccessful edge detection) and smoothed the data with an averaging time window of 10 frames (20 ms). We only focused on the time before the saccade execution because the effect of the gaze artifact on the apparent pupil size can be minimized when we evaluate the presaccade time range (Hayes and Petrov 2016). The presaccade normalized pupil size did not significantly differ between pro- and antisaccade tasks (Fig. 8*B*). Interestingly, however, there was a significant trial-by-trial correlation between elasticity coefficient of PSOp and the normalized pupil size within individual participants (Fig. 9). We found that a significant proportion of participants exhibited Spearman’s rank correlation coefficients larger than zero between normalized pupil size and the three parameters (Wilcoxon signed-rank test for Fisher’s *z*-transformed correlation coefficients: ρ = 0.088, *P* = 0.042 for overshoot amount, ρ = −0.069, *P* = 0.042 for viscosity coefficient, and ρ = 0.15, *P* = 0.0067 for elasticity coefficient; Fig. 9, *A–C*, *top*). As expected, there were no significant correlations between normalized pupil size and the viscoelastic parameters of PSOi (*P* values > 0.19; Fig. 9, *A–C*, *bottom*). This suggests that the observed correlations between normalized pupil size and PSOp may be caused by change in the physical property of the iris muscles, not by the characteristics dependent on the movement of the whole eyeball. It should be noted that in the present analysis, we calculated a partial correlation to assess the general relationship between pupil size and PSO, with the task effects partialed out. Given this fact and the result that the normalized pupil size did not show task dependence, we infer that the observed correlation between pupil size and PSOp may reflect the spontaneous fluctuation of the arousal level that varied from trial to trial during a block, rather than task-related factors.

Next, we evaluated the slope of the presaccadic trend of the pupil-iris diameter proportion. A previous study showed that the rate of the pupil size change is larger during the preparation for an antisaccade than during the preparation for a prosaccade (Wang et al. 2015). The slope values were calculated by the linear regression of the time course from −80 to −10 ms in Fig. 8*A* in a trial-by-trial manner. Consistently with the results by Wang et al. (2015), the pupil size change during the preparation for a saccade was larger for an antisaccade than for a prosaccade [*F*(1,13) = 9.46, *P* = 0.0089; Fig. 8*C*]. There was no significant main effect of modality (*P* > 0.11). The result indicates that involvement in the antisaccade task may enhance the pupil size change before saccade initiation regardless of task modality. A partial correlation analysis, which is the same as the above-described analysis of the absolute pupil size, showed no significant correlation between the slope of pupil size and PSOp/PSOi (*P* values > 0.13). This result suggests that the slope of presaccade pupil size change has little impact on the dynamic properties of PSOp.

#### Summary of mutual relationships among parameters.

**Fig. 10.**
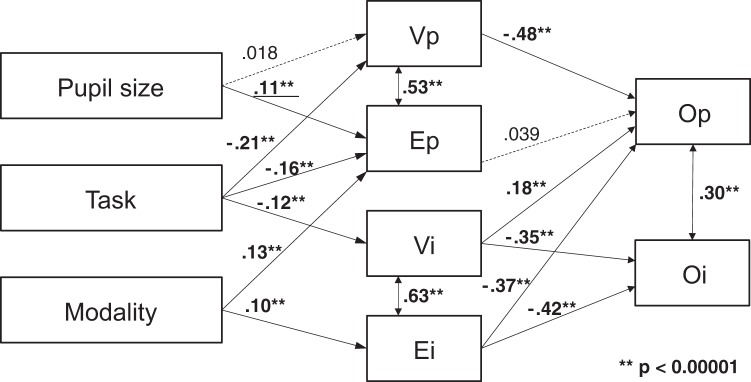
Path diagram visualizes the relationship between postsaccadic oscillation (PSO) parameters and the effects of task and pupil size (see text for details). The mutual relationship shown was derived based on trial-by-trial samples. V, E, and O represent the viscosity coefficient, elasticity coefficient, and overshoot amount, respectively; and p and i represent pupil-centric and iris-centric PSO, respectively. Consistent with the correlation analysis for each participant (Fig. 9), there was a significant causal relationship between pupil size and the elasticity coefficient of PSOp (*R* = 0.11).

To visualize the mutual relationships among the parameters examined so far, we next present the results of a path analysis. In advance of this analysis, we transformed the data into *z* scores within each trial block to rule out the effect of participant- and trial block-related components and excluded the trials with at least one instance of missing data in the parameters. Based on the results described earlier and the rationale behind the present study, we hypothesized the following paths: task should affect the viscosity coefficient of PSOp/PSOi (see the main effect of task, Fig. 5*A*) and the elasticity coefficient of PSOp (for the interaction between task and eye parts, see Fig. 6). Modality should affect the elasticity coefficient of PSOp/PSOi (see the main effect of modality, Fig. 5*B*). Pupil size should affect the elasticity coefficient of PSOp (correlation between presaccade pupil size and elasticity coefficient of PSOp, Fig. 9). The results of the path analysis (Fig. 10) indicated a significant causal relationship between pupil size and elasticity coefficient of PSOp (*R* = 0.11, *P* = 0.0000047), whereas the path between the pupil size and viscosity coefficient of PSOp was not significant (*R* = 0.02, *P* = 0.20). This result is consistent with the previous result, suggesting that the elasticity coefficient of PSOp may reflect the spontaneous changes in the state of pupillae muscles during a cognitive task. Consistent with the ANOVA results, the paths from the task to the coefficients of viscosity and elasticity were significant. We assumed that the coefficients of viscoelasticity should determine the overshoot amount calculated directly from the saccade trajectory. This was confirmed by a significant negative relationship between the coefficients of viscoelasticity of PSOi and overshoot amount of iris-centric trajectory. As to the amount of pupil-centric trajectory, there is a negative relationship between the viscosity coefficient of PSOp and overshoot amount, whereas the elasticity coefficient has no correlation with overshoot amount. We also found there is a positive relationship between the viscosity coefficient of PSOi and overshoot amount of pupil-centric trajectory.

It should be noted that the set of variables examined in this analysis was not able to explain sufficiently the changes in overshoot amount or viscoelasticity of PSO, as indicated by relatively low values of the goodness-of-fit index (0.80) and the adjusted goodness-of-fit index (0.53) of the model shown in Fig. 10.

#### Evaluation of distortion in camera image depending on saccade size.

**Fig. 11.**
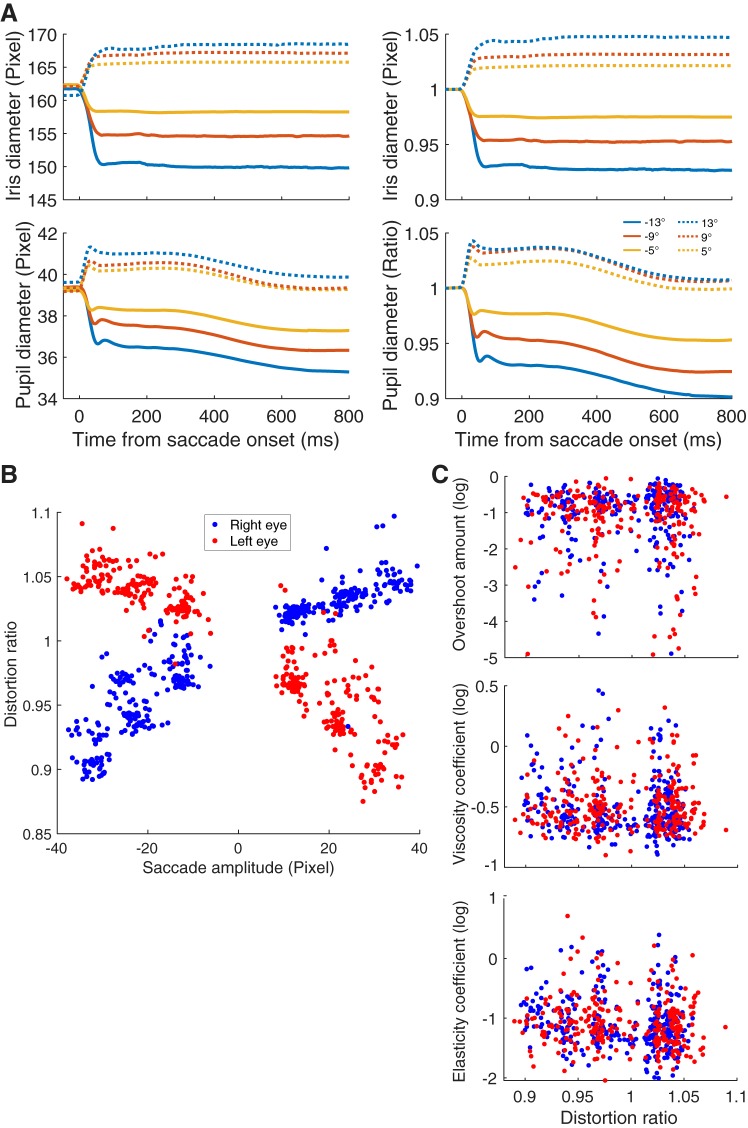
Evaluation of distortion in the camera image. *A*: time course of iris (*top*) and pupil (*bottom*) diameters during saccades. Only data for the right eye are shown. Diameters are represented as pixels in the camera image (*left*) and as the ratio of presaccade values (*right*). Because the right camera was placed on the right side of the right eye, the iris size appeared to decrease in the camera image for saccades toward the left stimuli (solid lines) and to increase for saccades toward the right stimuli (dashed lines). Pupil size generally decreased after the saccade. This was probably the pupillary light reflex driven by slight changes in light input due to the eye movements (Knapen et al. 2016). *B*: relationship between saccade amplitude and distortion ratio. There was a clear correlation, indicating that the distortion is caused by the angle between the eye gaze and the camera. *C*: relationship between distortion ratio and the properties of postsaccadic oscillation (PSO). Distortion in the camera image had no influence on the dynamic properties of PSO.

One may argue that distortions in camera images lead to serious artifacts for estimating the saccadic amplitude or other parameters. Such distortions could be induced by the three-dimensional–to–two-dimensional transformation when a camera captures eyeball images. We considered that iris size is a good indicator of the distortion in a camera image that occurs during a saccade because the iris size is physically unchanged. We defined the distortion ratio as the ratio of presaccade and postsaccade iris sizes (Fig. 11*A*, *top*). Compared with the iris size change, the pupil size dynamically changed around the time of saccade execution and the pupil constricted after several hundred milliseconds of saccade initiation (Fig. 11*A*, *bottom*). These phenomena probably occurred because PSOp is larger than PSOi and pupil constriction occurs after a saccade (Knapen et al. 2016). Figure 11*B* shows a clear relationship between the saccade amplitude and the distortion ratio, indicating that there is a gaze-dependent error in saccade amplitude estimation with a camera image. We then calculated partial correlations of the distortion ratio with saccade amplitude or PSO properties while controlling for the remaining variables. There was a strong (partial) correlation between the distortion ratio and saccade amplitude (Spearman’s correlation coefficient: ρ > 0.939, *P* < 10^−23^), indicating that the distortion was caused by saccade amplitude. Where this effect was controlled, there were no significant correlations between the three PSO parameters and distortion ratio (*P* values > 0.192; Fig. 11*C*). The overall results of *experiment 2* indicated that the distortion in the camera image can strongly affect saccade amplitude estimation but cannot account for the change in the PSO properties.

## DISCUSSION

This study aimed to clarify whether the pupil-centric postsaccadic oscillation (PSOp) and iris-centric PSO (PSOi) reflect cognitive processes such as an antisaccade task. Overall, we found that both PSOp and PSOi were larger for an antisaccade task than for a prosaccade task. The elasticity coefficient of PSO behaves somewhat differently from the viscosity coefficient or overshoot amount: the effect of a saccade task (pro vs. anti) on elasticity was not observed for PSOi but was observed for PSOp. This indicates that the mechanical properties of the iris muscles influencing the elasticity of iris wobble may vary depending on the cognitive task.

### 

#### Possible mechanisms for task dependence of elasticity coefficient of pupil-centric postsaccadic oscillation.

We found that the elasticity coefficient of PSOp was sensitive to the task, whereas that of PSOi was not, indicating that cognitive tasks may affect the state of the iris muscles. Possible candidates are the muscles controlling pupil size (sphincter/dilator pupillae muscles). In fact, as reported in a previous study, we confirmed that the pupil size change before saccade initiation was larger for antisaccade than for prosaccade (Fig. 8*C*; Wang et al. 2015). However, there was no significant correlation between the slope of the pupil size change and the elasticity coefficient of PSOp. Rather, we found a significant correlation between the presaccade absolute pupil size, which did not exhibit task dependence (Fig. 8*B*), and the elasticity coefficient of PSOp. In contrast, there were no significant correlations between presaccade pupil size and any of three dynamic properties of PSOi (Figs. 9 and 10). These results suggest that the spontaneous fluctuation in arousal level during a cognitive task, which probably changes the state of the sphincter/dilator pupillae muscles, influences the elasticity of iris wobble. Another possible source of this correlation is the neural link between SCi and the pupil control circuit (Wang et al. 2012; Wang and Munoz 2015): preparatory activity in SCi during the antisaccade task is reflected in pupil size, and this pupil size change may in turn change the dynamic properties of PSOp, but not those of PSOi. It should be noted that the contribution of pupil size to explain the iris wobble is small because the observed correlation between absolute pupil size and elasticity of PSOp was weak (*r* ≈ 0.1).

The interpretation of the relationship between pupil size and iris wobble is not straightforward. The constrictions of the dilator and sphincter muscles may affect the viscoelasticity of the iris tissue, which in turn influences PSOp. Pupil size indicates only the relative degree of constriction of the two muscles. Moreover, the sphincter/dilator pupillae muscles form a complex structure: the sphincter muscle encircles the pupil and contracts in a circular manner, whereas the dilator muscle changes in a radial manner around the sphincter muscle. Probably, the weak correlation may be due to this complexity.

Another factor that might contribute is the state of the ciliary muscles, which control lens accommodation. It is possible that the postsaccadic lens oscillation (cf. He et al. 2010; Tabernero and Artal 2014; Tabernero et al. 2016) indirectly affects the PSOp due to the close spatial proximity between the lens and iris. A previous study tried to test this hypothesis and reported that PSOp was larger at near viewing distances than far viewing distances (Nyström et al. 2015). However, we consider that the task dependence of PSOp was not likely due to the change in the state of the lens. This is because Nyström et al. also found that pharmacological manipulations of lens accommodation did not have consistent effects on PSOp. They suggested that the distance dependence of PSOp can be explained by a change in vergence angle rather than oscillation of the lens. In the present study, we did not manipulate the viewing distance.

#### Task dependence of iris-centric postsaccadic oscillation.

We found significant effects of the cognitive task on the overshoot amount and viscosity coefficient for PSOi as well as PSOp. The result for PSOi may indicate that the task dependence is revealed by the PSO of the whole eyeball movement, which is considered to be caused by neural control signal reversals (Bahill et al. 1975). Kapoula et al. (1986) suggested that dynamic overshoot occurs when the braking pulse is accidentally too large and it serves no useful purpose. Given this notion, its relation to the cognitive task found in the present study suggests that the braking pulse is larger for antisaccade than for prosaccade. A further neurophysiological experiment is needed to clarify the neural mechanism of the task-dependent change in PSOi.

#### Contributions of other factors.

One may suspect that the observed task dependence of the PSO is possibly an artifact of our particular choice of stimuli or recording system. For example, we presented filled squares as the target for saccades. This target was foveated at the saccade landing only for the V_pro condition. One should be reminded, however, that the antisaccade amplitude did not show task dependence in the visual cue condition. Moreover, the antisaccade effect on PSO was observed even for the auditory cue condition, where visual markers were always open squares. Therefore, it is unlikely that the detailed properties of the markers (i.e., filled squares vs. open squares) contributed to the present results.

Another potential artifact is related to the decentration of the pupil in accordance with its size, which is observed for pupil-based eye tracker signals (Choe et al. 2016; Nyström et al. 2016; Wyatt 2010). However, we reject this explanation. If this is correct, there should be a correlation between the distortion in the camera image and the PSO. In *experiment 2*, we confirmed that there is a clear relationship between distortion and saccade amplitude but not between distortion and PSO properties.

#### Implications for cognitive and clinical sciences.

The present study suggests that the separate measurements of pupil-centric and iris-centric signals allow us to examine whole eyeball movements and the iris wobble, which contains more or less independent information about the states of the eye. The possible usefulness of these detailed eye movement measurements to answer fine-grained questions about the oculomotor system was suggested in a previous study (Nyström et al. 2013). The present study extends this view and to our knowledge is the first to reveal the relationship with the cognitive state.

The general approach described in the present study can be readily applied to an analysis of microsaccades, which are involuntary small eye movements during fixation. Several studies have suggested that both saccades and microsaccades share a common oculomotor generator (Otero-Millan et al. 2008). It is known that microsaccades reflect various aspects of perception, attention, and cognition (Martinez-Conde et al. 2009, 2013; Rolfs 2009). The dynamics of microsaccades may serve as an additional way of probing the cognitive state via eye metrics.

A recent study showed that the PSOp is larger for older people than for younger people (Mardanbegi et al. 2018). This is unexpected given the previous study by Deubel and Bridgeman (1995), which showed that the size of the dynamic overshoot in dual Purkinje eye tracker (DPI) signals decreases with age. This discrepancy may be resolved by considering independent motion properties of the eyeball, iris tissue, and the lens. The oscillation in the DPI signal may reflect the motion of the lens, whereas that in the pupil-based eye tracker signal, measured using video (e.g., EyeLink), reflects iris wobble. The former method may capture the stiffness of the ciliary muscle or zonular fibers, and the latter method may capture the state of the pupillae muscles. In future studies, detailed analyses of eye movement, as performed in the present study, may account for the discrepancy and in turn reveal the effects of age on eye-movement control and related cognitive processes. Aging is known to affect antisaccade behavior, as well. It is reported that the performance of antisaccade increases with development and starts to decrease from 20 yr of age (Coe and Munoz 2017). Given the findings of the present study, future examinations of PSO may allow us to probe aging effects on internal processes relating cognition and the oculomotor system.

## DISCLOSURES

No conflicts of interest, financial or otherwise, are declared by the authors.

## AUTHOR CONTRIBUTIONS

S.Y., M.Y., and S.F. conceived and designed research; S.Y. performed experiments; S.Y. analyzed data; S.Y., M.Y., and S.F. interpreted results of experiments; S.Y. prepared figures; S.Y. and S.F. drafted manuscript; S.Y. and S.F. edited and revised manuscript; S.Y., M.Y., and S.F. approved final version of manuscript.
